# Urinary DKK3 as a predictor of CKD stages in reflux nephropathy in children

**DOI:** 10.55730/1300-0144.6215

**Published:** 2026-02-25

**Authors:** Ayşe Seda PINARBAŞI, Neslihan GÜNAY, İnayet GÜNTÜRK, Didem BARLAK KETI, Sibel YEL, Sekure Rabia ULUEREN, Cevat YAZICI, Hakan M. POYRAZOĞLU, İsmail DURSUN

**Affiliations:** 1 Division of Pediatric Nephrology, Department of Pediatrics, Eskişehir Yunus Emre State Hospital, Eskişehir, Turkiye; 2 Division of Pediatric Nephrology, Department of Pediatrics, Faculty of Medicine, Erciyes University, Kayseri, Turkiye; 3 Department of Nutrition and Dietitian, Faculty of Health Science, Osmaniye Korkut Ata University, Osmaniye, Turkiye; 4 Department of Biochemistry, Faculty of Medicine, Erciyes University, Kayseri, Turkiye; 5 Faculty of Medicine, Erciyes University, Kayseri, Turkiye

**Keywords:** Urinary DKK3, chronic kidney disease, children, reflux nephropathy

## Abstract

**Background/aim:**

Reflux nephropathy is an important cause of chronic kidney disease (CKD) in children. The search for new biomarkers continues, as early intervention is important to slow the progression of CKD. Urinary Dickkopf-3 (uDKK3), a potential marker of tubulointerstitial fibrosis, has been studied in adults but not extensively in children with CKD. This study was conducted to determine the role of uDKK3 levels in predicting the CKD stage in children with reflux nephropathy.

**Materials and methods:**

This cross-sectional study included 113 children with CKD stages 1–4 with reflux nephropathy and 28 healthy controls. uDKK3 concentrations were measured using enzyme-linked immunosorbent assay. The association between uDKK3, the estimated glomerular filtration rate (eGFR), and albuminuria was investigated. Receiver operating characteristic (ROC) curves were used to assess diagnostic performance.

**Results:**

uDKK3 levels were significantly higher in CKD stages 3 and 4 compared to stages 1 and 2 and the control group. A strong negative correlation was observed between uDKK3 and the eGFR, while a positive correlation was noted with albuminuria. ROC analysis revealed an area under the curve (AUC) of 0.77 for the uDKK3 to urinary Cr (uCr) ratio (uDKK3/uCr), with a sensitivity of 71.1% and a specificity of 71.4% at a cutoff value of 1675.48 pg/mg.

**Conclusion:**

The correlation between the uDKK3/uCr and CKD severity suggests that uDKK3 may serve as a potential noninvasive biomarker for assessing disease severity. However, its utility for identifying early-stage CKD could not be clearly demonstrated in this cohort. uDKK3 appears to be particularly associated with advanced CKD stages, and further large-scale prospective studies are needed to clarify its clinical applicability and prognostic value in pediatric CKD.

## 1. Introduction

Chronic kidney disease (CKD) is a significant global public health problem [1]. It is defined as abnormalities of kidney structure or function, present for more than 3 months, with implications for health [2]. Although registry data of different countries have shown different results, congenital anomalies of the kidneys and urinary tract (CAKUT) are the most common cause of CKD in children. Vesicoureteral reflux (VUR) is one of the most common CAKUT subgroups. Reflux nephropathy is characterized by renal scarring resulting from VUR and recurrent urinary tract infections and represents one of the leading causes of CKD in children. The most important sequelae of reflux nephropathy are hypertension and CKD [3–5]. Therefore, the diagnosis, follow-up, and surgical and medical treatment of reflux nephropathy are important.

Although the progression of CKD varies according to the underlying causes, individual differences may also have an impact on the course of the disease [1]. Serum creatinine (Cr) is currently the most common biomarker used to follow-up children with CKD and to determine the severity of disease. However, it is affected by age, race, sex, and muscle mass. Therefore, new biomarkers, such as neutrophil gelatinase-associated lipocalin (NGAL) and kidney injury molecule-1 (KIM-1), have been studied to predict the course of the disease [6–8]. These have been used in recent years as markers of acute tubular injury. However, their effectiveness in predicting the progression of CKD is controversial. A metaanalysis by Zhou et al. [8] showed that the urine KIM-1 level is an independent risk factor for CKD stage 3, and the urinary NGAL value is an independent predictor of CKD5. Measuring NGAL in urine is not commonly used in clinical practice due to difficulties in preanalytical and analytical measurement [6]. For these reasons, few studies have recently focused on urinary Dickkopf-3 (uDKK3), a newly defined marker of tubulointerstitial damage and fibrosis independent from underlying diseases [9].

Kidney fibrosis occurs due to endothelial mesenchymal transition and extracellular matrix deposition. Regardless of the reason for initial damage, it has been shown that DKK3 has a profibrotic effect on the kidney using Wnt signaling pathways [10–13]. Since DKK3 is released into the urine in response to tubular stress, it may serve as a short-term, noninvasive diagnostic biomarker of reduced estimated glomerular filtration rate (eGFR) [9].

Studies on the relationship between uDKK3 and CKD in humans are limited in the literature. Most were performed on adults with glomerular pathology [9,14,15] and demonstrated that the uDKK3 to urinary Cr ratio (uDKK3/uCr) is significantly higher in adults with CKD than in healthy controls and has a negative correlation with eGFR [9,10]. Therefore, it may be a useful biomarker for predicting short-term eGFR loss in patients with CKD [9]. This cross-sectional study was performed to determine the possible role of uDKK3 levels in predicting CKD stages in children with VUR. It was speculated that if DKK3 levels are high in the urine and increase parallel to the CKD stage, as in the study of Zewinger et al. [9], it might be used as a noninvasive biomarker for the follow-up of patients with CAKUT.

## 2. Materials and methods

This prospective study included children with CKD stages 1–4 with primary or secondary VUR at Erciyes University Faculty of Medicine, Pediatric Nephrology Clinic, between June 2020 and March 2021.

Since there were no pediatric studies at the time of study design, a literature search was performed to determine the number of patients to be included in the study. Based on the most extensive human analysis [9], the average uDKK3/uCr was 350 pg/mg, with a standard deviation (SD) of approximately 350 pg/mg in the control group. Assuming that this value would be at least two-fold higher in the patient groups (i.e. about 700 pg/mg), with an alpha of 0.50, and a power of 0.80, the minimum sample size for each group was calculated as 16 patients. According to the power analysis, at least 16 patients per group were required; however, 30 patients per group in stages 1–4 CKD and 30 healthy children of similar age and sex, as the control group, were planned to be included (150 in total). Inclusion criteria for the healthy volunteers were no history of recurrent urinary tract infection, hydronephrosis, or CAKUT. Patients using immunosuppressive drugs or those with symptoms and/or laboratory findings of concomitant symptomatic urinary tract infections were also excluded from the study.

Urinalysis, urine culture, urine albumin, protein, Cr, blood urea nitrogen (BUN), serum Cr, cystatin C, and complete blood count were measured in all participants. The urine albumin to Cr ratio (uACR) and urine protein to Cr ratio (uPCR) were calculated. The eGFR was calculated using the Schwartz, cystatin C, and Chronic Kidney Disease in Children (CKiD) formulas. The CKD groups were formed based on the eGFR calculated using the CKiD formula [16] and classified according to the Kidney Disease: Improving Global Outcomes (KDIGO) CKD-albuminuria classification [2]. Spot urine samples were collected and centrifuged at 370 *g* for 10 min to remove sediments and stored at −20 °C immediately for short-term storage or −80 °C for long-term storage for DKK3 analysis.

### 2.1. uDKK3 analysis

After thawing at room temperature, the urine samples were analyzed for Cr using the Jaffe reaction. uDKK3 measurements were performed using commercially available enzyme-linked immunosorbent assay (ELISA) kits (Catalog No: RAB0145; Sigma-Aldrich Chemical Co., St. Louis, MO, USA) that did not cross-react with other DKK proteins (DKK1, DKK2) or albumin, as detailed in the supplementary data.

### 2.2. Statistical analysis

All statistical analyses were performed using IBM SPSS Statistics for Windows 22.0 (IBM Corp., Armonk, NY, USA). p < 0.05 was considered statistically significant. The normality of the data was checked using the Kolmogorov–Smirnov test. Data with normal distribution were expressed as the mean ± SD, and parameters with nonnormal distribution were expressed as the median [interquartile range (IQR)]. Nonparametric tests were used because the group sizes were ≤30, and most of the variables were not normally distributed. Categorical variables were expressed as percentages and were analyzed using the Pearson’s chi-squared test or Fisher’s exact t test. The Kruskal–Wallis and Mann–Whitney U tests were used to compare abnormally distributed independent variables. The Spearman’s correlation analysis was used as the correlation test. In addition, we formed the receiver operating characteristic (ROC) curves. The area under the curve (AUC) was determined to calculate the sensitivity and specificity of the uDKK3/uCr for CKD. The diagnostic value of the uDKK3/uCr was evaluated using the ROC curves.

Comparisons of the uDKK3/uCr, uACR, and eGFR with healthy children of similar age and sex were performed. Since the uDKK3/uCr did not have a specific cutoff point, it was divided into tertiles, as previously reported [17], with an equal number of patients in each group (33.3rd and 66.6th percentiles: 1853.5 and 16728.7 pg/ng, respectively). The group with the highest uDKK3/uCr (tertile 3) was compared with the other two groups using the chi-squared and Kruskal–Wallis tests, and post hoc analysis was conducted. Using a backward stepwise model, multiple regression analysis was performed with the uDKK3/uCr as a dependent variable and the eGFR, hemoglobin, uACR, and weight standard deviation score (SDS) as independent variables.

Moreover, to evaluate albuminuria, the study group was divided into three subgroups based on the degree of albuminuria. The uACR was divided into three groups, as <30 mcg/g: normal–mild, 30–300 mcg/g: moderate, and >300 mcg/g: high-severity albuminuria [2]. Statistical analyses similar to those for the DKK3 groups were performed, except for the multiple regression analysis.

## 3. Results

The study included 113 patients with CKD stages 1–4 due to primary or secondary VUR and 28 healthy controls. As previously stated, it was planned to enroll 30 patients in each group. However, due to the COVID-19 pandemic, enrolling a sufficient number of patients in the stage 4 CKD group was not possible. Hence, patient enrollment comprised 30 with CKD stage 1, 33 with stage 2, 29 with stage 3, and 21 with stage 4.

The median age of the patients was 11.14 (6.76–14.12) years, and 54.9% were male. Primary reflux nephropathy was present in 68.1% of the patients. The median eGFR was 74 (33–90) mL/min/1.73 m^2^. The demographic and laboratory characteristics of the patients are shown in Table 1. The age, sex, uDKK3/uCr, and uACR distribution of the patients in each CKD group are shown in Table S1. The median uDKK3/uCr values of the control group and patients with CKD stages 1–4 were 1166, 1711, 1526, 17126, and 81821 pg/mg, respectively (Table S1).

**Figure 1:**
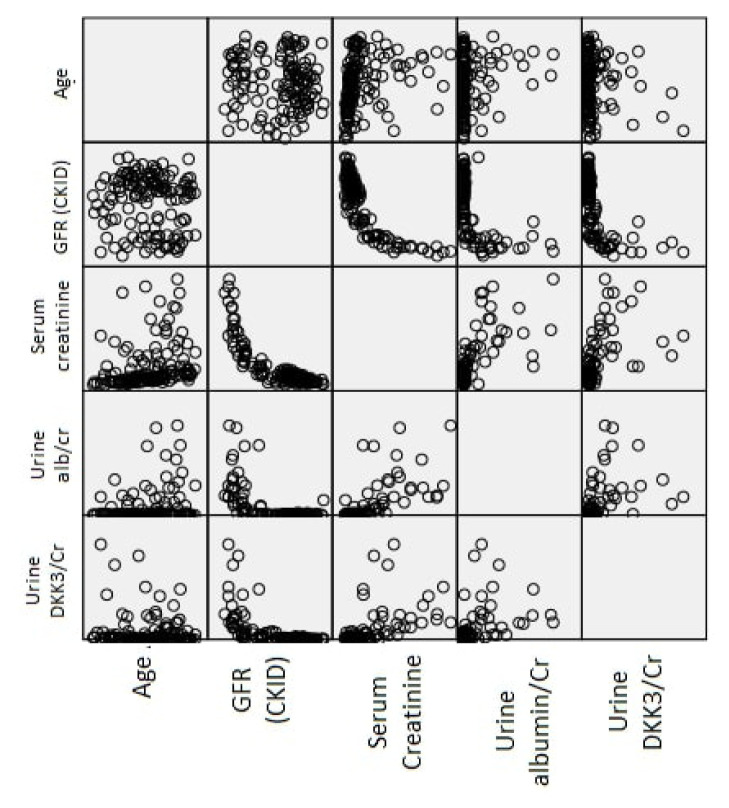
Correlation matrix of the uDKK3/uCr with important parameters.

The correlation matrix between the uDKK3/uCr and age, eGFR, serum Cr, and uACR is shown in Figure 1. The uDKK3/uCr was negatively correlated with the eGFR and positively correlated with the uACR. In the patient group, the uDKK3/uCr was negatively correlated with weight and height SDS values; however, in the control group, the uDKK3/uCr was not correlated with age, weight SDS, or body mass index (BMI) SDS values (data not shown).

**Figure 2:**
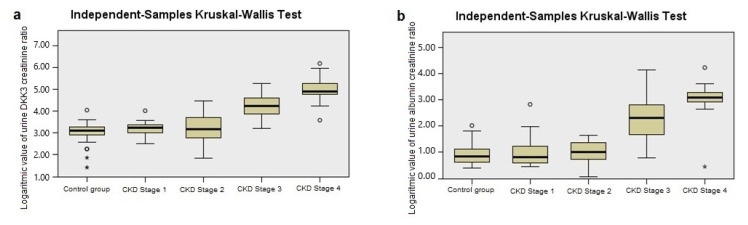
a) Logarithmic uDKK3/uCr values at different CKD stages, b) logarithmic uAlb/uCr ratio values at different CKD stages.

When the log values of the uDKK3/uCr and uACR were compared between the CKD groups and the control group, there were statistically significant differences between the control group and CKD stage 3 and 4 patients, between stage 1 and stages 3 and 4 patients, and between stage 2 and stages 3 and 4 groups. There was no significant relationship between the control group and stage 1 and 2 patients (Figure 2). The distribution of patients according to the KDIGO 2024 CKD-albuminuria classification [2] and similarly, the distribution of patients in the DKK3 subgroups are shown in Table S2. When the study group was divided into three groups according to the KDIGO 2024 CKD-albuminuria classification, the patients in the stage 4 CKD group were mostly in the severe albuminuria group. The uDKK3/uCr was also statistically significantly higher in the severe albuminuria group (p < 0.001) (Table S3).

When the spot uDKK3/uCr was divided into three groups with the same sample size, CKD stages 1 and 2 patients were mainly in DKK3 tertile 1, and CKD stages 3 and 4 patients were in DKK3 tertile 3. In line with this, the eGFR was statistically significantly lower in DKK3 tertile 3. The uACR and uPCR were much higher in DKK3 tertile 3 (Table 2).

Univariate regression analysis examining factors independently associated with the uDKK3/uCr is presented in Table 3. In the CKD group, while hemoglobin, weight SDS, albuminuria, and eGFR at baseline were independent predictors for an increased uDKK3/uCr, only baseline eGFR was an independent predictor for an increased uDKK3/uCr in the multivariate regression analysis (R2 = 0.532, p < 0.001).

**Figure 3:**
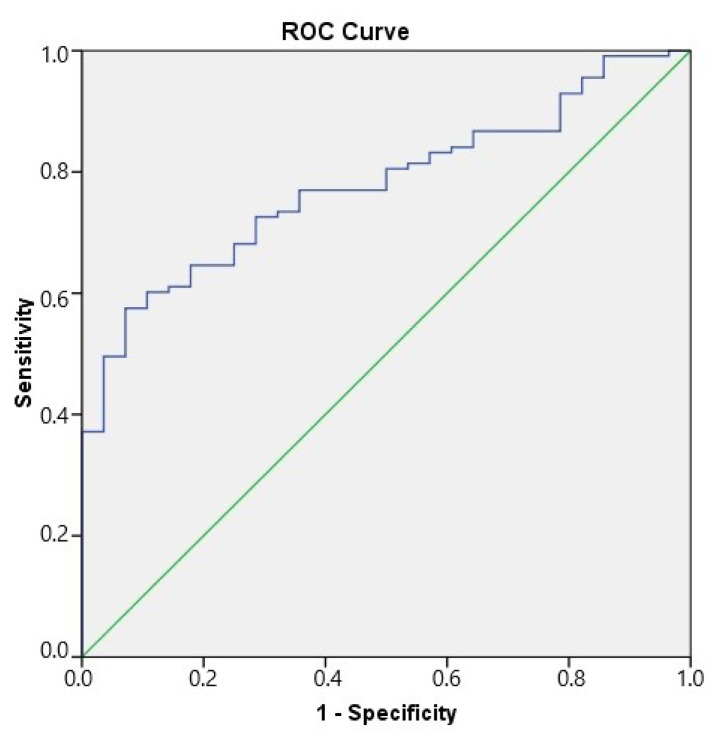
ROC curve for the uDKK3/uCr values of the participants, AUC: 0.778 (95% CI: 0.699–0.858), p < 0.01.

ROC analysis revealed that the AUC for the uDKK3/uCr was 0.77 (95% CI: 0.69–0.85). With a cutoff value of 1675.48 pg/mg, the sensitivity and specificity were 71.1% and 71.4%, respectively (Figure 3 and Table 4).

## 4. Discussion

This cross-sectional study investigated the potential of uDKK3 as a biomarker for different CKD stages in children with VUR. The findings suggest that the uDKK3/uCr is significantly elevated in children with higher CKD stages, particularly stages 3 and 4, compared to those with lower stages and healthy controls. This finding aligns with previous adult studies identifying uDKK3 as a potential marker of tubulointerstitial damage and fibrosis, and thus CKD, and suggests that it may also be useful in children.

The most comprehensive study about uDKK3 in kidney disease was conducted by Zewinger et al. [9], who evaluated the uDKK3/uCr in a healthy population and in patients with CKD stages 2–4 with a variety of underlying etiologies. They found that uDKK3 was elevated in patients with CKD, independent of underlying etiology, compared with the general population. They also showed that in biopsy specimens from patients with glomerular disease and interstitial kidney disease, elevated uDKK3/uCr was significantly associated with a higher degree of tubulointerstitial fibrosis [9]. In line with this, Federico et al. [10] conducted a study on 72 young patients, with a mean age 13.57 years, who had nephronophthisis and glomerulopathy, and analyzed urine samples for uDKK3 excretion and found that their uDKK3 levels were negatively correlated with the eGFR. They also studied a cohort of 36 adult patients with various types of degenerative and immunological glomerular and tubulointerstitial diseases, and showed that uDKK3 levels, but not serum Cr, correlated strongly with the degree of tubular atrophy and interstitial fibrosis seen in kidney biopsies. As the current study only included patients with CAKUT without an indication for biopsy, no comparison could be made between tubulointerstitial fibrosis and uDKK3.

It is known that proteinuria and albuminuria are important markers of CKD progression. For this reason, albuminuria was also included in the KDIGO guidelines, in addition to the eGFR [2]. An important study demonstrating the superiority of uDKK3 in this respect was conducted by Gonzalez et al. [18] on 118 diabetic patients with an eGFR of >90 mL/min were compared with 50 healthy control subjects. A biopsy was taken from diabetic patients with proteinuria, and the amount of interstitial fibrosis was compared with a number of urine-derived biomarkers. Among these biomarkers, after adjustment for age and proteinuria, only uDKK3 was associated with the degree of fibrosis. For this reason, uDKK3 is considered to be an important biomarker for the early detection of diabetic nephropathy. In the present study, a positive correlation was found between the uDKK3/uCr and albuminuria and proteinuria. As with diabetic nephropathy, early identification of reflux nephropathy may allow early surgical and medical intervention to reduce the progression of CKD. However, it is clear that more research is needed on the relationship between uDKK3 levels and proteinuria in the healthy population, as this has not been adequately investigated in the literature [13].

In the present cohort, the uDKK3/uCr in early CKD stages (stages 1 and 2) were comparable to those of the healthy controls. This suggests that while uDKK3 reflects disease severity in advanced CKD, its ability to identify early-stage disease could not be clearly demonstrated in this cross-sectional cohort. Larger studies including healthy children and patients with early-stage CKD are needed to clarify its role in early disease detection.

One of the most comprehensive and recently published studies on children with CKD showed that CKD progression was faster in patients with a uDKK3/uCr >1689 pg/mL and that GFR decline was faster in patients with a uDKK3/uCr increase >1000 pg/mL within 6 months, according to the evaluations made at 6-month follow-ups [19]. In the present study, the cutoff value for the uDKK3/uCr in the ROC analysis was 1675 pg/dL, with a sensitivity of 71.1% and a specificity of 71.4%. Since it was very close to the value of 1689 pg/dL that Speer et al. [19] stated was important for CKD progression, patients with reflux nephropathy who have a uDKK3/uCr above this value should be carefully examined for the need for surgical intervention. The uDKK3/uCr cutoff identified in the current cohort may reflect disease severity in children with reflux nephropathy. Its significant association with both the eGFR and albuminuria suggests that uDKK3 reflects ongoing renal injury and may provide supportive information alongside conventional markers. However, its independent prognostic value requires confirmation in prospective pediatric studies.

In a recent adult study, Sánchez-Álamo et al. [20] studied both patients with CKD of various etiologies (32% vascular, 19% diabetes mellitus, 10% glomerular, 10% polycystic kidney disease, 10% chronic interstitial nephritis, and 15% unknown) and a cohort with diabetic nephropathy to evaluate the possible role of uDKK3 as a predictor of long-term renal progression. Both cohorts had relatively long follow-up, with a median of 36 months. The primary endpoint was a >50% increase in serum Cr or end-stage kidney disease or death. The median uDKK3 levels were significantly higher in patients who achieved the primary endpoint of the study. Among the underlying causes of CKD, the median uDKK3/uCr was similar. They also found that elevated uDKK3 levels were a significant and independent predictor of CKD progression after adjustment for the eGFR and proteinuria. Herein, the uDKK3/uCr was independently associated with the eGFR, even after adjusting for other potential confounders such as body weight, weight SDS, and albuminuria.

However, the study had some limitations. The main one was that it coincided with the COVID-19 pandemic. Therefore, the study had to be cross-sectional. The relatively small sample size, especially in CKD stage 4, requires further research in larger cohorts to confirm these findings. To validate the use of uDKK3 as a reliable biomarker for CKD progression in children, future multicenter studies with larger sample sizes are needed.

Despite these limitations, this study provides valuable insights into the potential role of uDKK3 as a noninvasive biomarker for monitoring CKD progression in children with VUR. The significant association between the uDKK3/uCr and CKD stage highlights its potential as a marker of disease severity and progression; however, its utility for early-stage identification could not be clearly demonstrated in our cohort and should be evaluated in larger prospective studies. Further research is warranted to confirm these findings and establish the clinical utility of uDKK3 in pediatric CKD management.

## Conclusion

The findings herein, together with the existing literature, suggest that uDKK3 has potential as a noninvasive biomarker associated with CKD severity and progression in children with reflux nephropathy. However, its role as an independent marker for early-stage CKD could not be clearly demonstrated in our cohort. Given its association with eGFR decline and albuminuria, further large-scale prospective pediatric studies are needed to clarify its clinical utility and prognostic value.

## Supplementary data

### 1. Materials and methods

#### 1.1. uDKK3 analysis

The principle is based on the formation of antigen-antibody complexes without competition. DKK3 (unlabeled antigen) found in urine samples binds to antibody coated wells. The biotin-labeled antibody is then added. Following incubation of antibody–antigen-labeled antibody complexes (sandwich) with added enzyme (horseradish peroxidase (HRP)-Streptavidin) followed by substrate, the intensity of color increases in direct proportion to the concentration of DKK3 present in the sample.

For each kit, the DKK-3 standard graph was drawn with CurveExpert 1.4 using the absorbance values corresponding to the standard concentrations. uDKK-3 levels calculated from the graphics were given as pg/mL (Figure S1).

### 2. Results

The distribution of age, sex, uDKK3/uCr, and albumin/Cr ratios of the patients according to the groups are shown in Table S1.

The distribution of the patients according to the KDIGO 2024 CKD-albuminuria classification and similarly the distribution of the patients according to the DKK3 groups are shown in Table S2.

When the study group was divided into three groups according to the KDIGO 2024 CKD-albuminuria classification, the patients in the stage 4 CKD group were mostly in the severe albuminuria group. The uDKK3/uCr was statistically significantly higher in the severe albuminuria group (p < 0.001) (Table S3).

**Table S1:** Distribution of the groups and median values of important parameters.

Groups	N	Age (year) Median (25P–75P)	Male %	Spot urine	
				DKK3/Cr pg/mg Median (25P–75P)	Albumin/Cr mcg/mg Median (25P–75P)
Control group	26	10.8 (8.2–15.2)	53.8	1166 (757–1676)	7.04 (3.88–15.77)
Stage 1 CKD	30	10.0 (6.8–12.0)	50.0	1711 (977–2433)	6.52 (4.0–18.9)
Stage 2 CKD	33	10.4 (4.9–14.7)	48.5	1526 (561–5264)	10.63 (5.5–24.23)
Stage 3 CKD	29	11.9 (7.9–15.4)	55.2	17126 (6438–46312)	201.6 (46.2–703.3)
Stage 4 CKD	21	12.3 (7.5–14.6)	71.4	81,821 (50,142–22,7042)	1219 (802.5–2195.8)

**Table S2:** CKD classification according to the KDIGO 2024 GFR and albuminuria categories and DKK3 groups.

n = 109		Albuminuria, n (%)		
CKD stage	eGFR	A1	A2	A3
G1	≥90	24 (22.0)	3 (2.7)	1 (0.9)
G2	60–89	29 (26.6)	4 (4.5)	0 (0)
G3a	45–59	5 (4.5)	3 (2.7)	3 (2.7)
G3b	30–44	0 (0)	6 (5.5)	10 (9.1)
G4	15–29	0 (0)	1 (0.9)	20 (18.3)
n = 113		DKK3 group, n (%)		
CKD stage	eGFR	Group 1	Group 2	Group 3
G1	≥90	12 (10.6)	17 (15)	1 (0.9)
G2	60–89	16 (14.1)	13 (11.5)	4 (3.5)
G3a	45–59	0 (0)	6 (5.3)	6 (5.3)
G3b	30–44	0 (0)	2 (1.7)	15 (13.2)
G4	15–29	0 (0)	1 (0.9)	20 (17.6)

White: low risk, light grey: moderately increased risk, grey: high risk, dark grey: very high risk for CKD.

**Table S3:** Comparison of parameters according to albuminuria classification.

	Normal/mild Albuminuria <30 mcg/g n = 58	Moderate Albuminuria 30–300 mcg/g n = 17	Severe Albuminuria >300 mcg/g n = 34	P

Age (Year) [median (25p– 75p)]	10.4 (6.9–13.6)	9.4 (4.19–12.67) *	12.5 (8.3–15.3)	0.048

Sex [n (%)]				
Female	29 (50)	6 (35.3)	13 (38.2)	0.401
Male	29 (50)	11 (64.7)	21 (61.8)	

VUR [median (25p–75p)]				
Primary	52 (89.7)	11 (64.7)	11 (32.4)	<0.001
Secondary	6 (10.3)	6 (35.3)	23 (67.6)	

CKD [n (%)]				
Stage 1	24 (41.4)	3 (17.6)	-	<0.001
Stage 2	28 (48.3)	4 (23.5)	1 (2.9)	
Stage 3	5 (8.6)	10 (58.8)	13 (38.2)	
Stage 4	1 (1.7)	-	20 (58.8)	

eGFR [median (25p–75p)]				
Bedside Schwartz	100 (81–119) *	55 (43–106) *	23 (17.0–35.5)	<0.001
Cystatin C based	73 (64.5–79) *	46 (33–74.5) *	23 (20.5–32.0)	<0.001
CKiD formula	85 (75.5–96) *	53 (34–86) *	23 (20.0–34.0)	<0.001

Antropometric values [median (25p–75p)]				
Body weight SDS	–0.32 (–0.74–1.21) *	–0.71 (–1.05–0.09)	–1.33 (–2.43– −0.45)	<0.001
Body height SDS	–0.15 (–0.57–0.56) *	–0.52 (–1.45–0.76) *	–1.6 (–2.61– −0.72)	<0.001
BMI SDS	–0.19 (–1.0–0.96)	–0.31 (–1.15–0.49)	–0.72 (–1.57–0.62)	0.343

Laboratory parameters [median (25p–75p)]				

Serum Cr	0.59 (0.44–0.72) *	0.79 (0.44–1.25) *	2.24 (1.64–3.21)	<0.001

Hemoglobin	13.4 (12.7–14.05) *	12.6 (11.7–13.8) *	11.1 (10.4–12.65)	<0.001

CRP	0.84 (0.3–2.26) *	0.31 (0.18–3.05) *	2.43 (0.36–12.2)	0.010

WBC	7510 (6175–8605)	7700 (6305–8715)	7110 (6100–8285)	0.626

Sedimentation	6 (4–6.5) *	6 (5–12) *	24 (10.5–51)	<0.001

Spot urine				
Protein/Cr	0.15 (0.09–0.19) *	0.25 (0.20–0.46) *	2.04 (1.34–3.29)	<0.001
DKK3/Cr (pg/mg)	1678 (912–3009) *	7224 (2796–26,943) *	60,509 (16,910–109,924)	<0.001

DKK3 Groups [n (%)]				
Group 1 DKK3	32 (55.2)	4 (23.5)	-	<0.001
Group 2 DKK3	23 (39.7)	5 (29.4)	8 (23.5)	
Group 3 DKK3	3 (5.2)	8 (47.1)	26 (76.5)	

* Statistically significant when compared with the uALB/uCr group (post hoc analysis), CRP: C-reactive protein, WBC: white blood cell.

Figure S1 Standard calibration curve for the DKK3 ELISA generated by plotting optical density against serially diluted DKK3 standards. Linear regression analysis demonstrated excellent linearity within the working range (r = 0.9999), confirming the accuracy and reliability of quantitative DKK3 measurements. [X axis: optical density (OD); Y axis: concentration (pg/mL)].

## Figures and Tables

**Table 1:** Demographic characteristics and laboratory parameters of the patients.

Variables	Values

Age (year) [median (25p–75p)]	11.14 (6.76–14.12)

Male [n (%)]	62 (54.9)

Body weight SDS [median (25p–75p)]	−0.51 (−1.37–0.67)

BMI SDS [median (25p–75p)]	−0.36 (−1.34–0.76)

**Vesicourethral reflux**	
Primary [n (%)]	77 (68.1)
Secondary [n (%)]	36 (31.9)

**CKD**	
Stage1 [n (%)]	30 (26.5)
Stage2 [n (%)]	33 (29.2)
Stage3 [n (%)]	29 (25.7)
Stage4 [n (%)]	21 (18.6)

**GFR [median (25p–75p)]**	
Bedside Schwartz	80 (35–107)
Cystatin C based	60 (32–76)
CKiD	74 (33–90)

Serum Cr (mg/dl) [median (25p–75p)]	0.73 (0.47–1.65)

BUN (mg/dl) [median (25p–75p)]	14.1 (10.8 – 31.6)

Hemoglobin [median (25p–75p)]	12.8 (11.7–13.8)

**Proteinuria**	
uPCR (mg/mg)	0.218 (0.147–1.309)

**Albuminuria**	
uACR (mcg/mg)	25.50 (6.51–495.25)
Log albuminuria	1.40 (0.81–2.69)

**uDKK3/uCr [median (25p–75p)]**	
Spot urine (pg/mg)	3854.7 (1464.9–30246.3)
Log uDKK3/uCr	3.586 (3.165–4.480)

BMI: Body mass index, CKD: chronic kidney disease, BUN: blood urea nitrogen, uPCR: urine protein to creatine (Cr) ratio, GFR: glomerular filtration rate, uACR: urine albumin to uCr ratio, uDKK3: urinary Dickkopf-3, uCr: urinary Cr, uDKK3/uCr: uDKK3/uCr ratio.

**Table 2:** Comparison of the DKK3 subgroup parameters in the CKD patients.

	DKK3 Tertile 1 n = 37	DKK3 Tertile 2 n = 38	DKK3 Tertile 3 n = 38	P

Age (Year) [median (25p–75p)	12.2 (8.50–15.34)	8.18 (4.44–11.85)	11.8 (6.3–14.2)	0.002

**Sex [n (%)]**				
Femalea	22 (59.5)	16 (42.1)	13 (34.2)	0.081
Male	15 (40.5)	22 (57.9)	25 (65.8)	

**VUR n (%)**				
Primary	35 (94.5)	28 (73.7)	14 (36.8)	<0.001
Secondary	2 (5.5)	10 (26.3)	24 (63.2)	

**CKD [n (%)]**				<0.001
Stage 1	17 (45)	13 (34.3)	0 (0)	
Stage 2	18 (48.6)	12 (31.5)	3 (7.8)	
Stage 3	2 (5.4)	11 (28.9)	16 (42.1)	
Stage 4	0 (0)	2 (5.2)	19 (50)	

**eGFR [median (25p–75p)]**				
Bedside Schwartz	103.0 (87.2–120.7)*	95.0 (54.7–111.7)*	24.5 (17.5–42.0)	<0.001
Cystatin C based	74.5 (67.0–83.7)*	66.0 (48.5–77.5)*	27.0 (21–33)	<0.001
CKiD formula	87.5 (82.0–101.0)*	78.0 (52.0–91.7)*	25.5 (20.2–35)	<0.001

**Anthropometric values [median (25p-75p)]**				
Body weight SDS	−0.05 (–0.68–1.24)*	−0.72 (–1.26 – −0.28)	−1.2 (−2.5 – 0.11)	0.001
Body height SDS	0.09 (−0.43–0.84)*	−0.40 (−0.91–0.15)*	−1.56 (−2.41 – −0.77)	<0.001
BMI SDS	0.0 (−0.95–0.96)	−0.56 (−1.42– −0.04)	−0.38 (−1.52–0.69)	0.350

Laboratory values [median (25p–75p)]				

Serum Cr	0.6 (0.46–0.73)*	0.55 (0.40–0.88)*	1.98 (1.32–3.13)	<0.001

Hemoglobin	13.6 (13.0–14.27)*	12.65 (11.92–13.77)*	11.7 (10.5–12.8)	<0.001

**Spot urine**				
Protein/Cr	0.13 (0.09–0.18)*	0.21 (0.16–0.49)*	1.83 (0.49–3.32)	<0.001
Albumin/Cr	10.37 (4.59–22.06)*	10.32 (5.5–197.9)*	802.5 (100–1677)	<0.001

* Statistically significant when compared with “DKK3 Tertile 3” (Post Hoc analyses)

VUR: Vesicoureteral reflux, eGFR: estimated GFR.

**Table 3:** Univariate and multivariate analysis results for predicting the group with the highest uDKK3/uCr in children with CKD.

Univariate Regression				Multivariate Regression		
Valuables (n = 113)	OR	CI (%95)	p	OR	CI (95%)	p
eGFR (CKiD formula)	0.907	0.877–0.939	<0.001	0.912	0.874–0.952	<0.001
Hemoglobin	0.502	0.364–0.693	<0.001	0.829	0.468–1.468	0.520
uACR	1.003	1.001–1.004	<0.001	1.001	0.998–1.004	0.402
Body weight SDS	0.652	0.485–0.878	0.005	1.011	0.571–1.790	0.970
Age (year)	1.017	0.935–1.106	0.694			
BMI SDS	1.016	0.756–1.366	0.915			

**Table 4:** Area under the receiver operating characterictic (ROC) curve (AUC), sensitivity and specificity based on the optimized cutoff point for the uDKK3/uCr in CKD.

AUC (95%)	Cutoff	p	Sensitivity (%)	Specificity (%)
0.778 (0.699–0.858)	1675.48	<0.01	71.1	71.4

## Data Availability

Data will be supplied upon request.
