# Supplementary figures and images for: Urinary DKK3 as a predictor of CKD stages in reflux nephropathy in children
Source: Turk J Med Sci. 2026 Feb 25;56(3):808–16. doi: 10.55730/1300-0144.6215 (PMC13398555; doi:10.55730/1300-0144.6215)

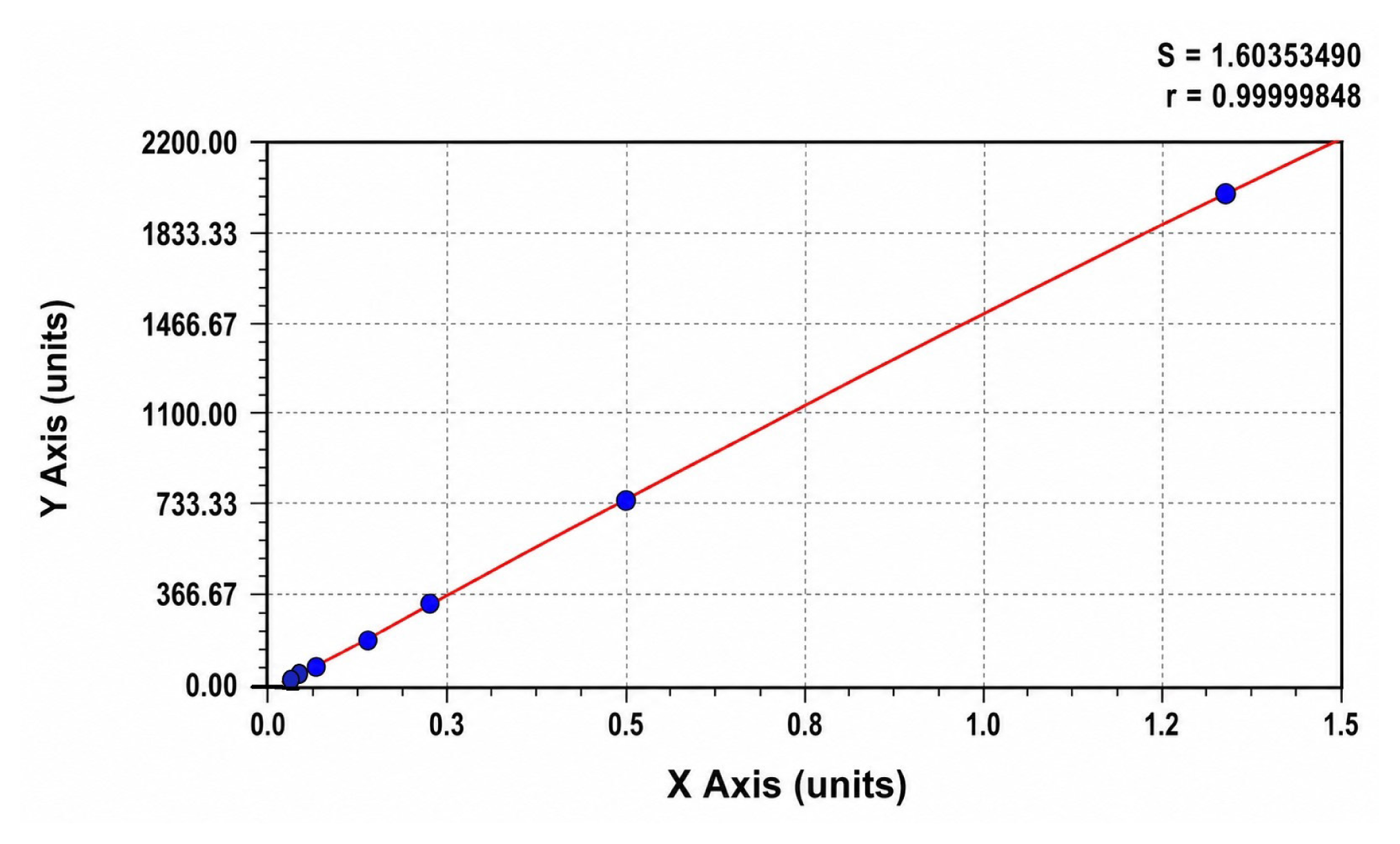

Supplement: Figure S1 — Standard calibration curve for the DKK3 ELISA generated by plotting optical density against serially diluted DKK3 standards. Linear regression analysis demonstrated excellent linearity within the working range (r = 0.9999), confirming the accuracy and reliability of quantitative DKK3 measurements. [X axis: optical density (OD); Y axis: concentration (pg/mL)]. [file tjmed-56-03-808s1.tif]
